# Objective Measures of Stress: Association of Speech Features and Cortisol

**DOI:** 10.1192/j.eurpsy.2025.616

**Published:** 2025-08-26

**Authors:** F. Menne, H. Lindsay, J. Tröger, A. König, M. Plichta, M. Schmidt-Kassow

**Affiliations:** 1 ki:elements GmbH, Saarbrücken, Germany; 2 Université Côte d’Azur, Centre Hospitalier et Universitaire, Clinique Gériatrique du Cerveau et du Mouvement, Centre Mémoire de Ressources et de Recherche, Nice, France; 3 Department of Psychiatry, Psychosomatic Medicine and Psychotherapy, University Hospital Frankfurt, Goethe-University Frankfurt am Main, Frankfurt, Germany

## Abstract

**Introduction:**

Stress is a physiological and psychological response that contributes to the development and worsening of psychiatric disorders such as depression and anxiety. Objective stress measurement is crucial in psychiatric settings, as stress affects the onset and progression of these conditions. Reliable data can improve diagnosis, treatment, and management. Speech analysis offers a non-invasive way to assess stress, as stress-induced physiological changes can influence features like pitch, jitter, and speaking rate.

**Objectives:**

This study aims to explore whether automatic speech analysis can serve as an objective stress measure by examining the relationship between speech features and cortisol levels during an acute stressor.

**Methods:**

Participants were recruited at the Department of Psychiatry, University of Frankfurt, Germany. Cortisol levels were measured in saliva before (T0) and 20 minutes after (T1) a stress-inducing or control task. Participants immersed their hand in cold or warm water while being observed via video, then completed a speech task by reading 16 standardized sentences before and after the task. Various speech features, including frequency, energy, and spectral characteristics, were analyzed in relation to cortisol levels. Correlations and mixed-effects models were calculated.

**Results:**

A total of 52 participants (n=28 stress, n=24 control) read 1040 sentences across T0 and T1. Cortisol levels increased in both sexes in the stress condition compared to the control (Figure 1). Vocal tremor showed a strong positive correlation with cortisol at T0 and a strong negative at T1 regardless of condition. The harmonic-to-noise ratio had no correlation at T0 but displayed a negative one at T1. Pitch range showed no initial correlation but was strongly negative post-stress. Mixed-effects models revealed significant interactions between time point and group for features like number of pauses and loudness standard deviation (p=.036 and p<.001, respectively). Loudness rate was significantly associated with time point (p<.001). In the linear mixed-effects model, an interaction effect was observed between time point and group for the harmonics-to-noise ratio, with a significant decrease in the stress condition (p<.001).

**Image 1:**

**Figure:**
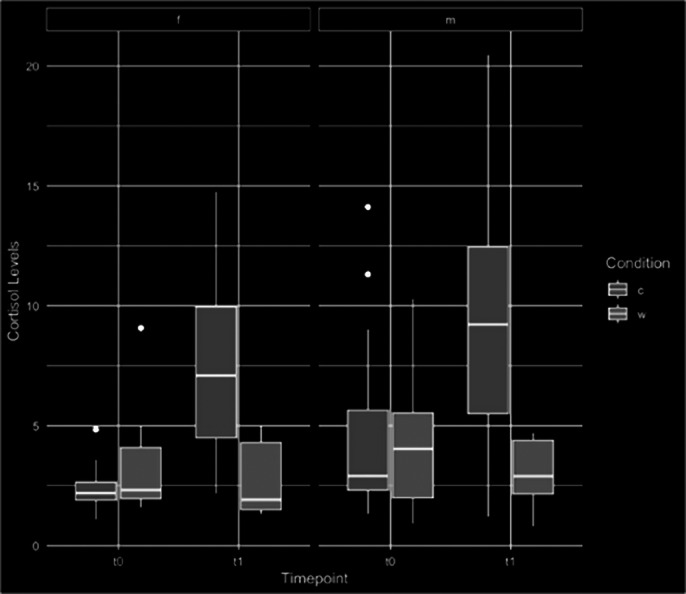

**Conclusions:**

This study supports speech analysis as a potential objective stress measure. Findings suggest that features like vocal tremor and pitch range are sensitive to acute stress, indicating that speech analysis could provide a non-invasive, real-time tool for assessing stress in psychiatric settings, offering an alternative to traditional self-report methods.

**Disclosure of Interest:**

None Declared

